# Provisioning an Early City: Spatial Equilibrium in the Agricultural Economy at Angkor, Cambodia

**DOI:** 10.1007/s10816-021-09535-5

**Published:** 2021-09-23

**Authors:** Sarah Klassen, Scott G. Ortman, José Lobo, Damian Evans

**Affiliations:** 1 Archaeological Sciences, Leiden University, Leiden, The Netherlands; 2 Department of Anthropology and Institute of Behavioral Science, University of Colorado Boulder, Santa Fe Institute, Santa Fe, USA; 3 School of Sustainability, Arizona State University, Tempe, USA; 4 École française d’Extrême-Orient, 22 Avenue du Président Wilson, 75116 Paris, France

**Keywords:** Agricultural intensification, Low-density urbanism, Settlement scaling theory, Urban land use, Angkor, Southeast Asia

## Abstract

A dominant view in economic anthropology is that farmers must overcome decreasing marginal returns in the process of intensification. However, it is difficult to reconcile this view with the emergence of urban systems, which require substantial increases in labor productivity to support a growing non-farming population. This quandary is starkly posed by the rise of Angkor (Cambodia, 9th–fourteenth centuries CE), one of the most extensive preindustrial cities yet documented through archaeology. Here, we leverage extensive documentation of the Greater Angkor Region to illustrate how the social and spatial organization of agricultural production contributed to its food system. First, we find evidence for supra-household-level organization that generated increasing returns to farming labor. Second, we find spatial patterns which indicate that land-use choices took transportation costs to the urban core into account. These patterns suggest agricultural production at Angkor was organized in ways that are more similar to other forms of urban production than to a smallholder system.

**Supplementary Information:**

The online version contains supplementary material available at 10.1007/s10816-021-09535-5.

## Introduction

One of the critical questions regarding the emergence of urban systems is how the farming fraction of the population generated sufficient food surpluses to feed a growing non-food-producing fraction (Adams, 1966; Trigger, 2003). This question is especially salient for Angkor, Cambodia, one of the most spatially extensive preindustrial urban agglomerations documented by archaeology. Recent research using a combination of airborne and spaceborne remote sensing techniques alongside ground surveys has revealed an urban area extending over at least 1500 km^2^ and a total population of up to 900,000 people, including more than 150,000 people in the densely populated core (Klassen, et al., 2021; Stark, 2004). The surprisingly high population of the urban center implies substantial surplus production by the farming fraction of the population. The need for considerable surplus production suggests that as the overall population of Angkor grew, outputs per farmer must have also increased substantially to feed a growing proportion of non-farming inhabitants. How was this accomplished?

The transition from a subsistence economy to an urban economy must involve an increase in food output per farmer and likely also an increase of output per unit of land to feed a growing number of non-producers in the urban centers. This process is known as agricultural intensification, and research in economic anthropology has proposed various mechanisms through which it can occur. In this paper, we address two shortcomings we perceive in the literature concerning this process. First, previous studies have tended to assume that agricultural production generally involves diminishing marginal returns—all other things being equal, output per farmer will decrease as the labor dedicated to farming increases (Barkley & Barkley, 2013; Geertz, 1963; McNall, 1933). According to this view, farmers must increase the cropping frequency (reduce fallow periods), increase their hours of work, or improve farming technology through better farming implements, or additional inputs of fertilizer or irrigation water, to counteract this process (Boserup, 1965; Morrison, 1994; Netting, 1993; Stone, 2001). We add our voice to that of Morrison and others (Lansing, et al., 2017; Morrison, 2006:85; 2007) in emphasizing that the social and spatial organization of production itself is an additional mechanism through which productivity per farmer can be increased, and we illustrate a specific mechanism through which this can occur. Second, there is a tradition of treating agricultural production separately from other forms of economic activity, labeling the former as the “subsistence economy” or “human ecology” and the latter as “craft production and exchange.” We will argue that concepts developed in economic geography and urban science apply equally well to both forms of production. We will make this case through the following analysis of the agricultural economy at medieval Angkor.

The concept of emergence plays an important role in our analysis. Emergence occurs when a system exhibits properties or behaviors that its constituent parts do not display. The salient system properties or behaviors emerge as a result of the parts interacting with each other, locally and globally (Miller & Page, 2007). We find evidence for emergent properties of the agricultural economy at Angkor at two different scales that are rooted in the phenomenon of spatial equilibrium, whereby individuals seek to balance the costs and benefits of their daily activities by adjusting their locations and movements. Although the empirical indications of this process are different at the community vs. metropolitan scales, our analyses suggest the same fundamental process underlies both sets of results. Below, we first provide evidence that agricultural production was organized at the scale of the temple community, similar to the supra-household level of coordination of irrigation by temples in Bali (Lansing, et al., 2017). We find that agricultural production exhibited increasing returns to scale (*i.e.*, increasing productivity per capita with an increasing labor force) by concentrating farmers, and their interactions in productive processes, in space. Second, we present evidence of spatial patterns in the properties of productive units with distance from the urban core that are consistent with standard models of urban land use for contemporary cities, especially the trade-off between transport costs and land values. Based on these results, we suggest that studies of agricultural production in early urban systems would benefit from increased attention to the social and spatial organization of agricultural production, and an approach that integrates agricultural and non-agricultural production under a common analytical framework.

### Background

The dominant view of agricultural intensification in anthropology can be traced to the work of Esther Boserup (1965). Boserup was an economist who argued that as regional population density increases, farmers can counteract increasing land pressure by becoming more productive, either by working more hours, increasing soil inputs, reducing fallow periods, and adopting more efficient farming implements and practices. Quantitative studies of contemporary smallholder systems in Sub-Saharan Africa generally document these effects: increasing population density leads to not only increased inputs of fertilizer but also decreased farm sizes and income per farm (Demont, et al., 2007; Josephson, et al., 2014; Muyanga & Jayne, 2014; Ricker-Gilbert, et al., 2014; Stone, 1996). Comparative studies from other parts of the world have found that improvements in technology can also help increase production (Bray, 1994). For example, ethnographic documentation suggests land and labor productivity is much higher for irrigated rice than for swidden rice (Hunt, 2000), and intercropping with legumes increases maize yields to a greater extent than reduced fallow (Pacheo-Cobos, et al., 2015). These studies seem consistent with the Boserupian tradition, which is grounded in a model of the farm family that seeks to maintain its living standards in the face of increasing regional population density.

There is a vast literature that debates the merits of Boserup’s model for the archaeological record, much of it concerned with the extent to which population vs. non-demographic factors are primary drivers and the degree to which her assumptions regarding economic agents apply cross-culturally (Conelly, 1992; Johnston, 2003; Morrison, 1994; Morrison, et al., 1996; Netting, 1990; Padoch, 1985; Stoner, 2017). Although a review of this literature is beyond the scope of this paper, we find that it has tended to overlook the social and spatial organization of agricultural production itself as an important factor in production. In other forms of production, aggregate labor productivity generally increases as larger numbers of individuals integrate their specialized productive efforts through a division of labor. Such groups can increase their collective skill, which is facilitated through “learning by doing” (Arrow, 1962), and their efficiency, which is enabled by reduced task switching (Arrow, 1994). The organization of farm labor itself is thus an additional option for increasing the marginal returns to labor by facilitating specialization and “knowledge spillovers” through which farmers learn from each other (Solow, 1997). Studies of smallholder production have noted that certain aspects of agricultural labor are organized at the supra-household scale (see, *e.g.*, Stone, 1996). Still, it is our impression that the *quantitative* effects of social organization have tended to be overlooked in studies of agricultural intensification.

The effects of social organization are especially salient for rice agriculture, which is much more labor-intensive than other dryland crops like wheat. Talhelm and English (2020) have found that rice farming societies tend to have strong social norms of reciprocity because of the need to coordinate water use and keep track of labor contributions. A classic case of supra-household organization of agricultural production is the traditional rice-growing communities of South and Southeast Asia, with the Balinese *subak* (“water temple”) being an especially famous example (Lansing, 1987; Lansing & Kremer, 1993; Lansing, et al., 2017).

**Fig. 1:**
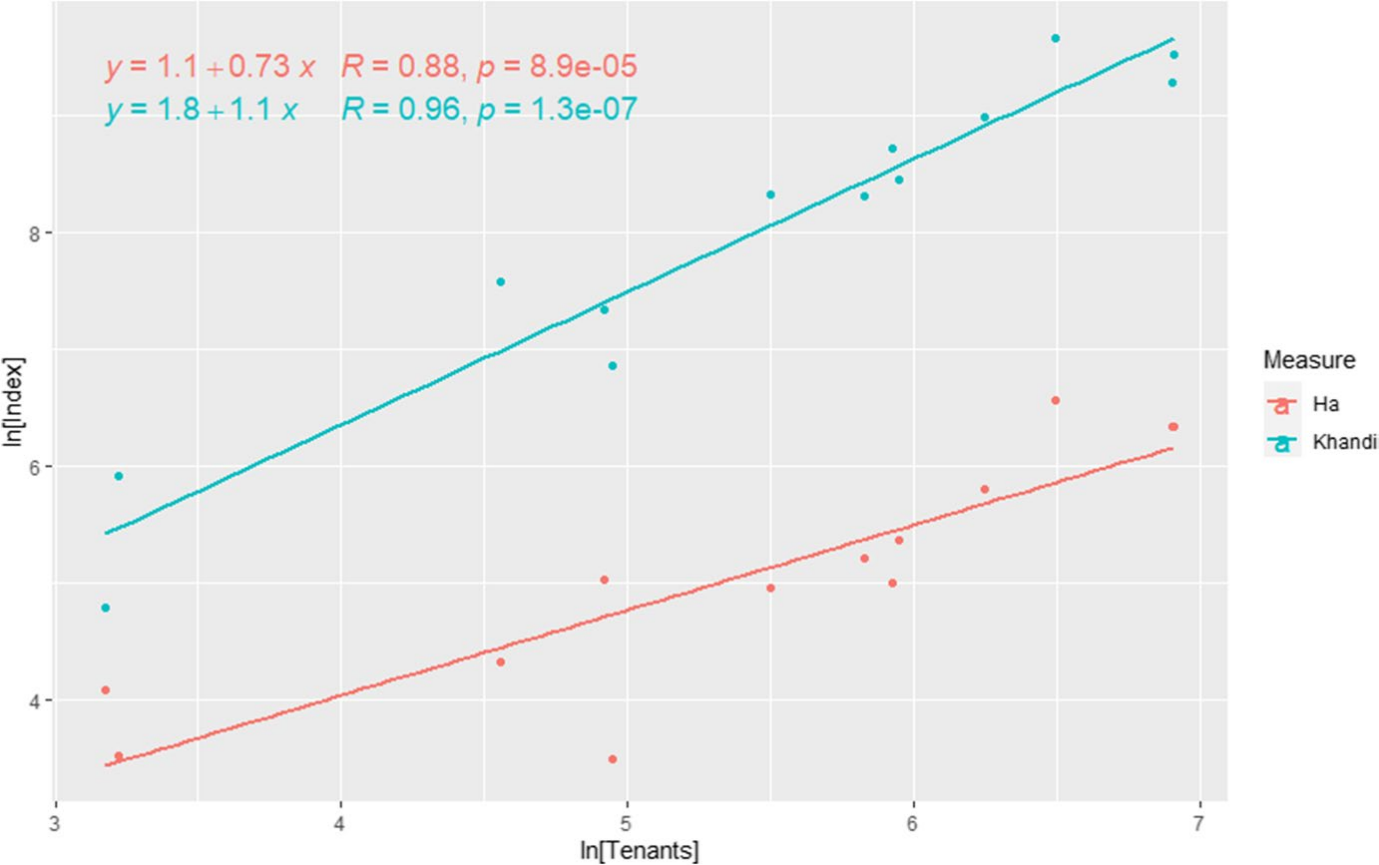
Relationships between farming labor (number of tenants), land (ha in production), and outputs (khandi, a unit of weight) in communidade rice paddies in Goa, India, ca. 1965. The coefficients of the regression equations (slopes of the fit lines) indicate decreasing land per farmer, but increasing output per farmer, with increasing community population. Data are from Axelrod and Fuerch (2006): Table 2

Intensive rice production involves complex irrigation infrastructure that allocates water to enable individual paddies to produce multiple crops per year. It is also highly labor-intensive in that it requires transplanting seedlings and plowing and managing flows of irrigation water. Intensive rice cultivation is well-suited to community-scale organization using a division of labor, and there is some evidence that such organization can generate increasing returns. Figure 1 presents ethnographic data collected from rice-growing *comunidades* in the Indian state of Goa, ca. 1965 (Axelrod & Fuerch, 2006). The population measure is the number of tenants who worked the rice paddies of a particular landholder, the area measure is the hectares of rice paddy in production in that community, and the output measure is the khandi (1 khandi = 266 pounds) of rice produced by that group per year. The two data series show that *comunidades* with more farmers produced greater output per person in a given year, even as they cultivated fewer hectares per person. These data provide direct documentary evidence that coordinated labor in intensive rice cultivation can produce increasing marginal returns. Below, we examine the extent to which the archaeological record of intensive rice cultivation at Angkor provides similar evidence.

Contemporary agricultural economics recognizes the effect of coordinated effort for increasing marginal returns to labor (Barkley & Barkley, 2013:41). Still, economic anthropology has tended to overlook the importance of labor coordination for net farming outputs, perhaps because practitioners have focused on smallholder systems where both land and labor inputs are organized primarily (though not exclusively) at the household level (*e.g.*, Netting, 1993). Nevertheless, increasing the marginal productivity of agricultural labor is the most important factor for producing the surpluses necessary to support a growing non-farming population.

Although we are not the first to emphasize the impact of social organization for agricultural intensification, studying it in an archaeological context is difficult because it requires observations of changing technological inputs, land, labor, and outputs over time and space, and these are often difficult to observe archaeologically. Angkor is unusual in this respect because proxy measures for all inputs to production are available. First, extensive remote surveys of the region using satellite and aerial imagery, supplemented by two campaigns of airborne lidar, have led to complete mapping of temple communities and associated agricultural infrastructure surrounding the civic-ceremonial core (CCC) (Evans, 2007, 2016; Evans & Fletcher, 2015; Evans, et al., 2013). Second, recent studies (Klassen & Evans, 2020; Klassen et al., 2018) have developed methods for dating temple communities using semi-supervised machine learning algorithms, resulting in a chronology of six periods tied to the reigns of significant rulers (Klassen, et al., 2021). These results, in combination with spatial associations of the mapped features, allow us to construct measures of land, labor, and inputs for agricultural production through time. In this paper, we examine the development of the agricultural landscape over these six periods, but with a focus on the final period, when the number of non-food producers in the CCC reached its peak.

### The Greater Angkor Region

Angkor is a prototypical example of *low-density agrarian urbanism*, a characteristic feature of which is farming within the settlement area itself (Fletcher, 2012). At Angkor, this included household vegetable gardens within the civic-ceremonial centers (CCCs) (A. Carter, et al., 2018; Castillo, et al., 2020) and over 1000 km^2^ of ricefields in the Angkor Metropolitan Area (AMA) that extended throughout the settled area (Hawken, 2012). Studies of documentary records indicate that agricultural production across the AMA revolved around temples organized communally and owned by elites, many of which had access to extensive state-sponsored hydraulic infrastructure (Klassen & Evans, 2020; Lustig & Lustig, 2019). Archaeological and inscriptional evidence indicates that these temple communities were the center of life for the medieval Khmer, controlling water and agriculture, managing a tenant labor force, and contributing to state coffers (Hall, 1985, 2011; L.A. Sedov, 1967). The nature of the labor force associated with these temple communities is unclear. Inscriptions describe large numbers of laborers as unfree or slaves (Lustig & Lustig, 2013, 2015), but there are also references to cyclical workgroups that would work 2 weeks of the month (Sahai, 2012; Stark, 2015).

In urban economics the term *metropolitan* has come to mean functional urban agglomerations united by common labor markets and infrastructure (Mehrotra, et al., 2020), but we use AMA to define the area beyond the CCCs that consist of community temples with reservoirs and ricefields. We argue that, together, the CCC and AMA form a single urban unit because the two are connected through an infrastructural network of reservoirs, canals and roads, which was likely used for periodic commuting, and the CCC cannot stand alone without the AMA (A. K. Carter, et al., 2021). Population densities in the CCCs reached as high as 38 people per hectare during the twelfth and thirteenth centuries CE, while the population density of the AMA hovered between 1.1 and 1.7 people per hectare from 889 to 1300 CE (Klassen, et al., 2021).

An account from Zhou Daguan, a Chinese diplomat who visited Angkor in 1296–1297 CE, states that Angkor could produce three to four rice crops per year, despite strong seasonality in precipitation (Daguan, 2007). Some scholars have argued that he referred to seasonal rhythms of production across the landscape rather than multi-cropping. There is also debate concerning the degree to which the massive hydraulic works at Angkor were involved in multi-cropping (Bourdonneau, 2010–2011). Although we do not subscribe wholesale to Groslier’s (1979) “hydraulic city” hypothesis, under which the huge (~ 16km^2^) barays (formal reservoirs) played a central role in feeding hundreds of thousands of people, it is nevertheless clear that various scales of hydraulic infrastructure—including a vast and intricate network of ricefield walls and embankments (Hawken, 2012, 2013), hundreds of smaller-scale dams, and many thousands of local community ponds—were used to carefully and deliberately manage the flow of water around the Greater Angkor Region (Evans, et al., 2007). This infrastructure, which required supra-household and supra-community cooperation for construction, maintenance, and proper functioning, was crucial for managing the delicate balance of water levels that are important for wet rice cultivation (Pottier, 2000). Donations of cows and buffalos to new temple foundations are mentioned in inscriptions (Jacob, 1993). It is likely that these bovine were used for animal traction and many ox-cart pathways have been documented among ricefields on the ancient landscape (Hawken, 2012).

The extensive landscape of interspersed fields and residential areas, linked by hydraulic and transport infrastructure, supported a population of over 150,000 in the urban core (Klassen, et al., 2021), which is known to have been the center of civic-ceremonial life and the location of numerous specialized workshops (A. Carter, et al., 2018; Daguan, 2007; Polkinghorne, 2012; Polkinghorne, et al., 2013). It is thus possible to distinguish between food-producing populations spread across the AMA on the one hand and an urban core population that did not produce food, beyond household gardens, on the other (Klassen, et al., 2021). Although the growth of the food-producing population outpaced the growth of the urban population for most of Angkor’s history, the final period, from 1181 to 1300 CE, marks a rapid expansion of population in the urban core. This period corresponds to the reign of Jayavarman VII, generally regarded as the apogee of the empire, after which a centuries-long period of decline set in (A. K. Carter, et al., 2021; A. K. Carter, et al., 2019).

Current data from Angkor suggest it had a significant non-food-producing population and that agricultural production was integrated into the overall system of social organization and physical infrastructure for water management and transportation. Given this situation, theoretical considerations reviewed below suggest that two patterns should be apparent. First, we should see evidence for increasing returns in agricultural outputs to temple community population, with an elasticity (growth of output relative to the growth of population) that is consistent with other forms of urban production (Bettencourt, 2013; Bettencourt, et al., 2007; Glaeser & Gottlieb, 2009). Second, the properties of agricultural temple communities should vary with distance from the urban core in ways that are consistent with models of urban land use, which primarily involve the effects of transport costs (Alonso, 1964; Brueckner, 1987; von Thünen, 1966). We find patterns consistent with both expectations in the data. Together, this evidence suggests the large-scale organization of agricultural production within the area of urban infrastructure is the primary mechanism through which Angkor was able to sustain itself (Fletcher, 2012).

## Materials and Methods

**Fig. 2:**
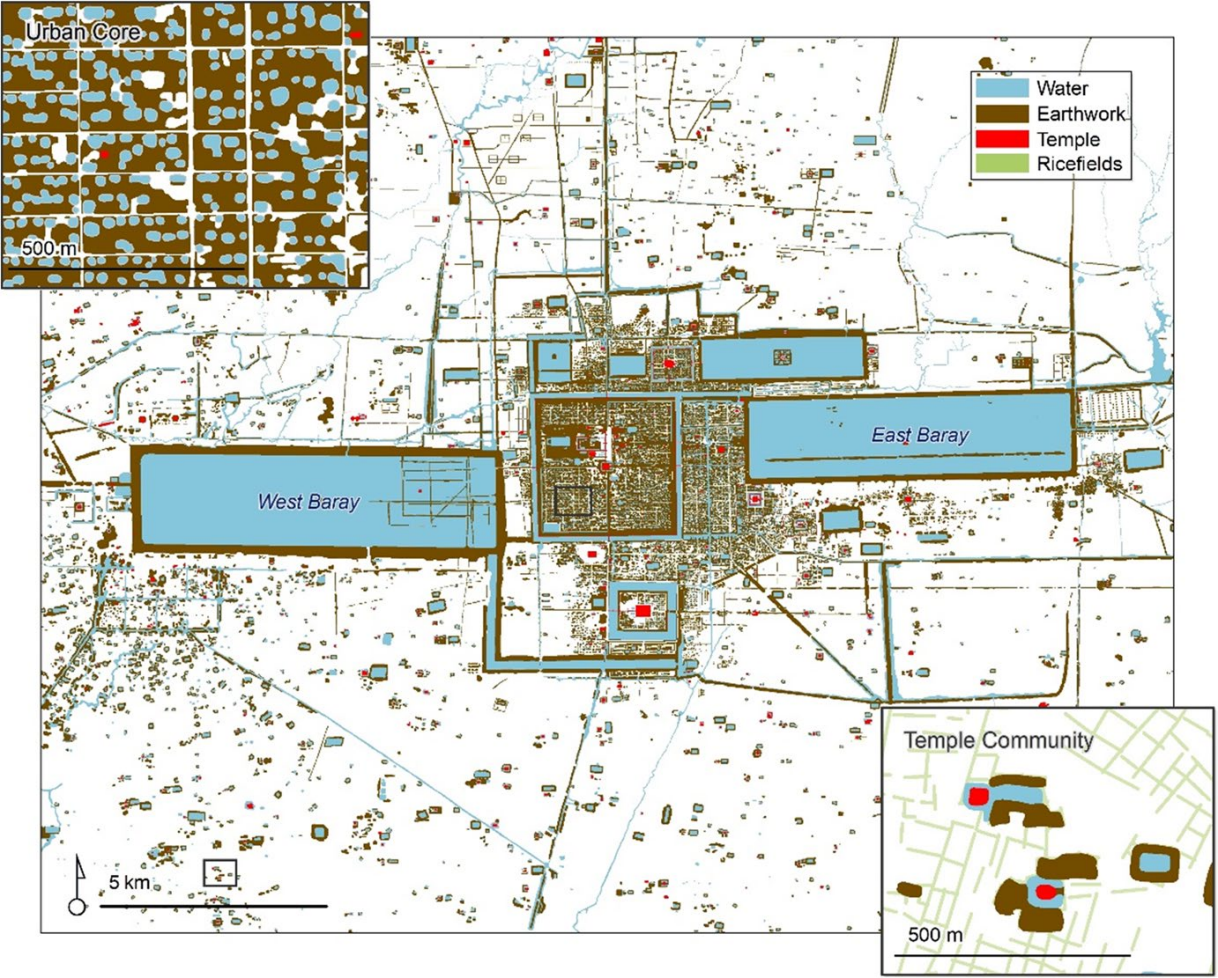
Map of Angkor, illustrating the densely occupied urban core surrounded by an extensive lower-density agricultural hinterland composed of temple communities. The temple platforms are red, occupation mounds are brown, moats and reservoirs are blue, and rice paddy bunds are green (inset only). Note that very little of the landscape was undeveloped by temple communities. Data courtesy of Damian Evans, Scott Hawken, and Christophe Pottier

**Fig. 3:**
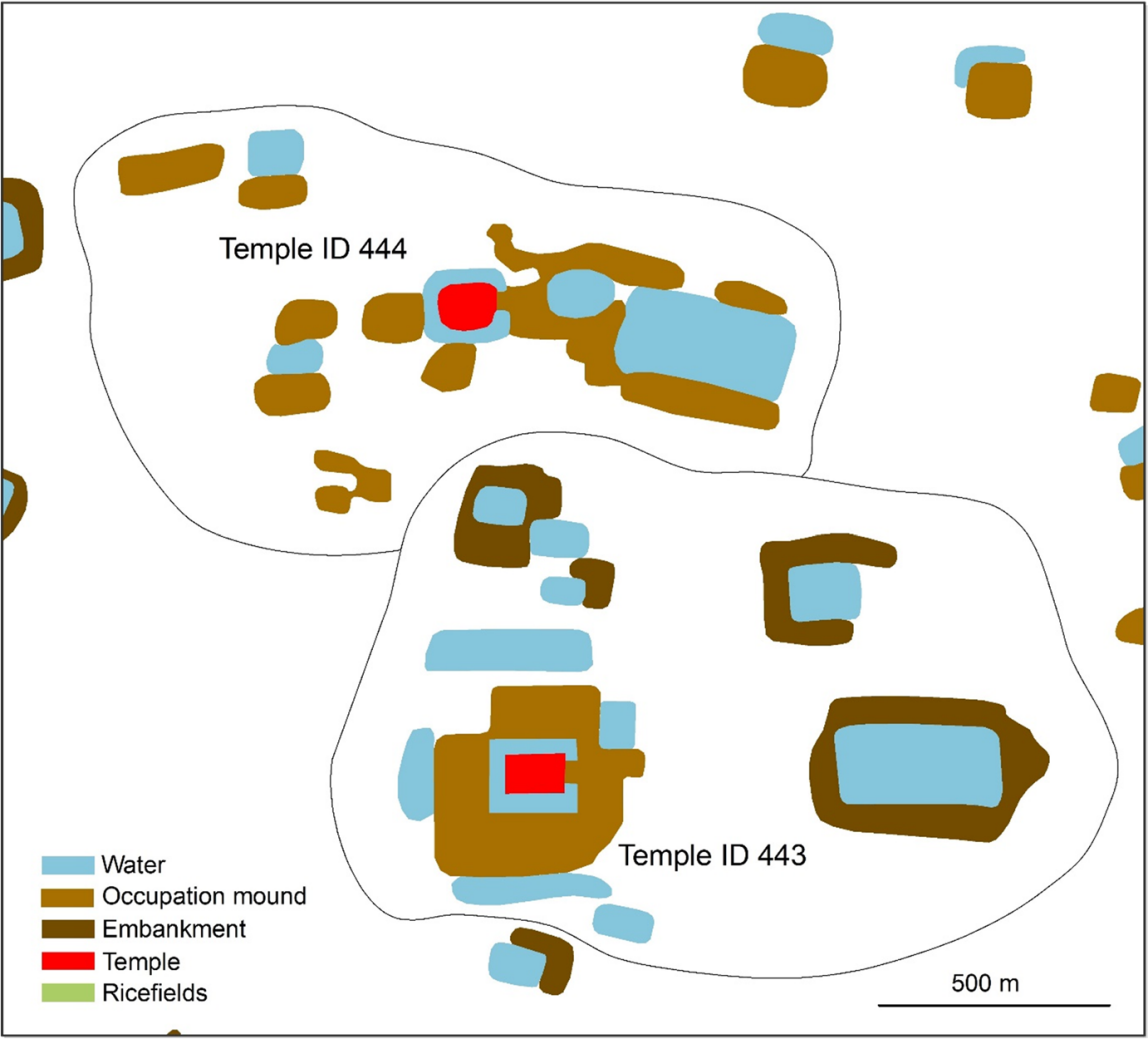
Map of Angkor, illustrating the extensive lower-density agricultural hinterland composed of temple communities. The temple platforms are red, reservoir embankments are brown, occupation mounds are light brown, moats and reservoirs are blue, and rice paddy bunds are green. This figure depicts two temple communities in particular: 443 and 444. Temple ID 443 was dated to 1132 CE and Temple ID 444 was dated to 865 CE using a combination of multiple linear regression and graph-based semi-supervised machine learning with an average absolute error of 49–66 years (Klassen et al., 2018). Temple ID 443 has a predicted population of 497 while Temple ID 444 has a predicted population of 338 (Klassen et al., 2021). Reservoirs were associated with each temple based on their orientation and proximity (Klassen et al., 2021). Based on our calculations, Temple ID 443 has a total holding capacity 108,807 m^3^ of water and Temple ID 444 has a total holding capacity of 23,415 m^3^ of water. Data courtesy of Damian Evans, Sarah Klassen, Pelle Wijker, Scott Hawken, and Christophe Pottier

In 2012, the Khmer Archaeological Lidar Consortium (KALC) acquired lidar data over 370 km^2^ of the Greater Angkor Region. These data revealed details of the urban form in the CCC and high-resolution (mm vertical resolution) topographical information about the region (Evans, et al., 2013). Combining this lidar imagery and other forms of remote sensing, including satellite imagery, researchers from the Greater Angkor Project have identified over 25,000 archaeological features in the Greater Angkor Region, including 1,000 temples, 9,000 reservoirs, and 9,000 mounded areas used for occupation. In addition, the temples and occupation mounds have been ground verified, and the reservoirs are very clearly identifiable in the remote sensing imagery. Figures 2 and 3 illustrate these features in the context of the Greater Angkor Region.

The basic unit of agricultural production in the AMA was the community temple, which many scholars have argued were centers of activity for medieval Khmer farming communities (Hall, 2011; Lustig & Lustig, 2019; L. A. Sedov, 1963; L.A. Sedov, 1967). These temples consist of an elevated rectilinear area of civic-ceremonial buildings, often surrounded by a moat, and associated residential areas and hydraulic farming infrastructure (Evans, et al., 2007). Researchers have been able to associate reservoirs and embankments with specific temples based on orientation and proximity (Klassen, 2018; Klassen, et al., 2021). In total, 46% of reservoirs (99% of reservoir water) and 92% of moats (28% of moat water) have been associated with specific temples in the AMA (see Table 3).

Recent advancements in statistical methods, including machine learning, have allowed researchers to date community temples and their associated moats and reservoirs with an average absolute error of 49–66 years (Klassen et al., 2018). These data were then combined with other archaeological data, association algorithms, art historical analysis, and radiocarbon dates to predict dates for features associated with temple communities in the AMA and archaeological features in the CCC. In total, researchers have been able to predict dates for over 20,000 of the 25,000 archaeological features that have been mapped in the Greater Angkor Region (Klassen et al., 2021). When combined with insights from inscriptions (Lustig & Lustig, 2019), this has led to a reasonably well-understood history of the agricultural landscape over time (Klassen & Evans, 2020). We carried forward these chronological assessments of community temples in this analysis. We acknowledge that the dating is subject to revision as more data are collected; however, we believe the level of chronological control we have achieved is sufficient for the present study.

For this analysis, we exclude features from the CCC and focus on agricultural temple communities from the surrounding AMA. To determine which reservoirs, moats, and temples are in the AMA, we used the Clip tool in ArcGIS 10.7.1 to remove features within CCC zones as defined by Klassen et al. (2021) from consideration. For total feature counts in the AMA and CCC, see Table 2.

### Estimating Temple Platform, Reservoir, and Moat Volumes

The digital terrain model (DTM) derived from the lidar data allows us to estimate the volume of water in reservoirs and moats and the volume of earth moved to create temple platforms and reservoir embankments for those features with lidar coverage. We calculated the depth and height of these features using the Zonal Statistics tool in ArcGIS 10.7.1 to the nearest cm. Then, we used the Range function to calculate the maximum value from the DTM raster minus the minimum value of the DTM raster inside of each moat, reservoir, mound, and temple polygon. We argue that the maximum and minimum values represent the original form of the features before they experienced erosion over the last 1000 years.

Once we calculated the depths of features within the lidar coverage, we used ordinary least-squares regression to measure the direction and strength of any relationship between feature depth and surface area. Temples with stone architecture (with heights > 2 m) were excluded from the regressions (Table 1). We then applied this equation to estimate depths (and thus volumes) based on the surface area of features within the Greater Angkor Region but not included in the 370 km^2^ zone of lidar coverage (Table 2).

**Table.1:** Depth regressions for features in the CCC and the AMA

Feature type	Sample size	*R*^2^	Regression equation	Total features
Reservoir	6161	0.027	*y* = − 0.0873 + 0.263*ln(*x*)	9,876
Reservoir embankment	509	0.325	*y* = 0.377 + 0.199(*x*)	2,817
Moats	224	0.3459	*y* = − 2.43 + 0.578*ln(*x*)	922
Temples	150	0.043	*y* = 1.43 + 0.0000685(*x*)	1009*

^*^Of these, 921 have predicted dates

**Table.2:** Features in the CCC and the AMA

Feature type	AMA	CCC
Reservoirs		
*Total count*	4,625	5,253*
*Total volume*	16.16 km^3^	0.28 km^3^
Moats		
*Total count*	789	133
*Total volume*	0.02 km^3^	0.004 km^3^
Temples		
*Total count*	749	262
*Total area*	2.09 km^2^	0.89 km^2^

* The large number of reservoirs, yet low water volume, in the CCC is reflective of the large number of house ponds which are shallower and have smaller surface areas than the larger reservoirs in the AMA associated with temple communities

### Estimating Labor Inputs

Labor inputs are based on the estimated populations of each temple community from Klassen et al. (2021). These estimates are based on the method developed by Hanson and Ortman (2017), which uses settlement scaling theory to translate the areas of more than 9,000 occupational mounds to estimates of their resident populations. Importantly, this method assumes that larger occupational mounds are more densely inhabited on average. These models were calibrated using ethnographic information concerning the typical population size of Khmer farming communities. We set the minimum population size of an occupational mound to one household of five persons. Each mound was then associated with the closest temple, and estimated populations of those mounds were summed to estimate that temple community’s population. For temples that no longer had surviving occupation mounds, we assigned an estimate of 497 people per community based on our indexing of mound areas to ethnographic information concerning the typical population size of Khmer farming communities (Delvert, 1961). Since an unknown fraction of these mounds has been destroyed for subsequent (post-Angkor) agricultural purposes and development, the preserved occupational mounds reflected in Table 3 provide a minimal estimate of food-producing temple community populations.

**Table.3:** Reservoirs and moats that were grouped with temples in the Angkor Metropolitan Area (AMA)

	With associated temple	No associated temple
Reservoirs		
*Total count*	2163	2462
*Total volume/area*	16.0 km^2^	0.16 km^2^
Moats		
*Total count*	687	102
Total volume/area*	0.0055 km^2^	0.0144km^2^

^*^The large number of reservoirs, yet low water volume, in the CCC is reflective of the large number of house ponds which are shallower and have smaller surface areas than the larger reservoirs in the AMA associated with temple communities

We recognize that farmers would have also contributed to other ritual, political, and economic activities. However, we argue that the amount of effort each farmer put toward agricultural production was independent of scale, so comparing outputs by scale capture the effects of scale irrespective of the other activities of these individuals.

### Estimating Land Inputs

The Greater Angkor Region is often defined as the 3000 km^2^ regional watershed. This area included large undeveloped patches in the North between Angkor and the Kulen mountains. Of the 3000 km^2^, Figs. 2 and 3 illustrate that approximately 1500 km^2^ of the land in the AMA was developed by community temples (Evans, 2007; Pottier, 1999), and over 1000 km^2^ of Angkorian period ricefields have been mapped over that 1500 km^2^ area (Hawken, 2012). This finding suggests it is reasonable to estimate the associated land in production for each temple community based on distances to adjacent temples during the period the temple was constructed and in subsequent periods as additional temple communities filled in the landscape. The land included in this analysis includes space for occupation, ricefields, and reservoirs. There does not appear to be a stark contrast between agriculturally productive and non-productive spaces as occupation mounds often included gardens (Castillo, et al., 2020), reservoirs were a source of fish protein, and inscriptions provide provisions to allow pigs to forage in ricefields (Jacob, 1993), all of which is included in our calculations of productivity.

Construction periods for agricultural temples are from Klassen et al., (2018), as discussed above. We have used these dates to assign temples to each of the six periods, ranging from 37 to 136 years. However, we acknowledge that there is likely to be some error in the period assignment of individual temples due to the absolute average error of 49–66 years in the dating method itself.

To calculate the amount of land available to each temple community, we estimated the transverse dimension of each community’s territory as the mean of the distance from each of the 921 dated temples in the dataset to its three nearest neighbors, based on their center-point locations. We squared this distance to estimate the total hectares of land within this territory. This method assumes temple community territories were generally rectilinear in outline. Still, it does not presume any specific geometry or even that the associated territory was centered on the temple. This method also avoids some of the boundary issues associated with alternative methods of defining site catchments, such as buffers or Thiessen polygons. It allows temple territories to vary continuously based on distances to neighboring temples.

Historical records indicate that land-associated community temples, and presumably their associated infrastructure, were often purchased and combined with pre-existing temples or used toward the foundation of new temple communities (Jacob, 1993; Lustig & Lustig, 2019). Although we cannot map these purchases, approximately 10% of new temples constructed during each period are within 250 m of another temple, based on the coordinates of their center points. Because temples and their associated moats were typically 40–80 m across, this implies a gap of less than about 150 m, which seems too close to reflect adjacent independent communities. We, therefore, consider such cases to represent the purchase of an existing temple by a new owner. To take this into account, we aggregated the data (including populations, territories, reservoir volumes, and total productivities) for all occupied temples within 250 m of one another during a given period, assigned these data to the most recently constructed temple in that group, and recalculated the community center location as the average of the contributing temple locations.

While we do not have comprehensive datasets that can be used to definitively model the terminus of occupation across our study region, emerging evidence suggests community temples and their associated infrastructure remained in use from the time of their dedication through the end of our study period in the fourteenth century CE, which is often referred to as the “collapse” of the Angkor. For example, in an extensive survey across the Greater Angkor Region, researchers have identified post-Angkorian ceramic scatters on occupation and temple mounds that indicate continued occupation through the post-Angkorian Period (Brotherson, 2019), while surveys within Siem Reap town that suggest the temples were used until at least the seventeenth century CE (Vitou, 2012). Similarly, Heng and Stark excavated three temple communities (Wat Athvea Area, Wat Chedei Area, Wat Prei Phdau Area) and found that each site had a long and complex occupation history with ceramics that date from the foundation of the temples until the 15th–twentieth centuries CE (Heng & Stark, 2020). Ultimately, any fallibilities with the assumption that all temple communities are occupied after their foundation to the end of the study period are not expected to impact our results because proportionate changes in the parameters of the model would not change any observed scaling patterns. This means that if only a percentage of the temple communities or occupation mounds are active during a given period, the scaling relationships should remain the same as there are no adjustments in parameters that co-vary with the total area of occupation mounds.

We estimated temple territories separately for each period based on all temples that had been constructed by that time unless a temple’s land and infrastructure met our criteria for an inferred purchase. Thus, for Period 1, territories are based only on temples constructed during that period, but Period 2 territories are based on temples constructed during Period 1 or 2, Period 3 territories are based on temples constructed during Periods 1–3, and so forth. The distribution of territories for all temples occupied during each period is presented in Fig. 5D. This chart shows that median temple territories fell from more than 500 hectares during Period 1 to about 200 hectares by Period 3 and a mere 140 hectares by Period 6. Also, the cumulative distributions show that new temple territories became less variable in size over time. Both are strong indications of increased packing of farming communities on the landscape, suggesting agricultural intensification.

### Estimating Irrigation Inputs

Agricultural intensification involves producing more rice per hectare per year. One of the ways to do this is to increase irrigation inputs. This can be estimated for temple communities based on the volume of water that could be stored in associated reservoirs and moats. In addition to moats surrounding temples, more than 3,000 reservoirs, which were generally constructed above grade, have been mapped for the Greater Angkor Region. During medieval times, water stored in these features was collected from precipitation, groundwater, local runoff, or state hydraulic infrastructure. Today, these ponds, called *trapeang*, are still used for crops and vegetables cultivated on their edges, but their use for rice cultivation is more limited (Pottier, 1999, 125–133). During medieval times, these reservoirs likely had several uses, including fish production, drinking water for animals, and as a source of irrigation water to flood rice paddies during the dry season (thus increasing the number of crops).

We summed the estimated volumes of all reservoirs and moats associated with each temple based on their proximity and orientation (Klassen, 2018; Klassen, et al., 2021). This measure can be associated with a temple during its construction period, assuming that reservoirs were built based on conditions at that time, or during the final period, under the assumption that reservoirs reflect the final distribution of temples and their associated lands. This volume can be considered a measure of the irrigation inputs to the land associated with a temple community. It can also be considered a measure of the infrastructure for agricultural production utilized by the associated community. At this stage, it is not possible to determine whether reservoirs were added to temple infrastructure as their territories shrank over time or were part of the initial development of a temple community. A program of OSL dating of reservoir embankments might help to answer this question.

### Estimating Agricultural Outputs

It is typical in economics to estimate the output of a social group as a product of multiple inputs (Robinson, 1953). In traditional agriculture, these inputs are land, labor, and technology, which includes farming equipment and inputs such as fertilizer, insecticide, and irrigation water (Hunt, 2000). Technology can be interpreted broadly to include know-how. Under ideal circumstances, we would have independent estimates of total outputs in addition to land, labor, and technology for a subset of temple communities. In the absence of such information, we multiply our measures of farming labor, agricultural land, and irrigation inputs to create a measure of temple agricultural output. We consider this three-factor measure to be more realistic than a single-factor measure, such as land by itself, due to the opportunities for multi-cropping provided by hydraulic infrastructure. If two of the three factors were held constant (land and water), one would expect the third factor (labor) to exhibit decreasing returns, but in a context where all three are free to vary, one would expect their product to be proportional to total production and to potentially exhibit increasing returns.

**Fig. 4:**
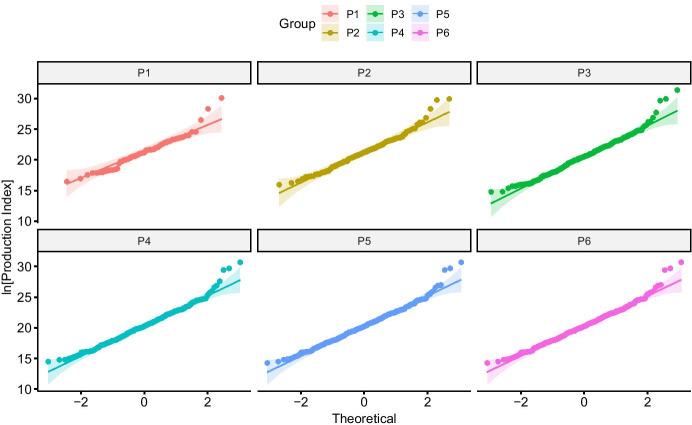
QQ plots of temple community agricultural output measures, by period

Because agricultural production can generally be viewed as resulting from the strong interaction of several factors of production (*i.e.*, land, labor, and other inputs), one would expect the probability distribution of a multiplicative production index to be log-normal (Aitchinson & Brown, 1957; Limpert, et al., 2001). Figure 4 presents QQ plots for the distributions of log-transformed production indices across temple communities during each period, assuming cumulative occupation. These plots show that these distributions all conform to the expected range of a log-normal distribution except for a few outliers in the upper tails.

### Relationship Between Community Scale and Productivity

As discussed above, rice paddy production in South and Southeast Asia was often organized at the local community scale, with groups of laborers coordinating agricultural activities. Under such conditions, one would expect the group’s productivity to increase proportionately with the interactions between workers per unit time and each worker’s effort. This is typical of non-agricultural socioeconomic outputs (Bettencourt, 2013; Bettencourt, et al., 2007; Glaeser & Gottlieb, 2009), but to our knowledge, this relationship has not been considered in previous studies of agricultural intensification. Furthermore, an interaction can involve exchanging physical things or information from one person to another during production. In addition to saving time, one would expect these interactions to improve each worker’s skill through emulation of best practices and social learning (Boyd, et al., 2011). At Angkor, these varied social interactions would have occurred within the developed network of physical spaces that include temples, rice paddies, ditches, and reservoirs.

A foundational assumption in the study of socioeconomic production embedded in physical space is that the benefits resulting from social interactions are balanced against the costs of the movement necessary for these interactions to occur (Isard, 1956). An approach to human networks known as *settlement scaling theory* builds from this principle to generate expectations for the effect of population size for interaction rates and outcomes. Importantly, agents do not seek to maximize outputs or minimize costs in this framework but simply seek a balance between movement costs and interaction benefits, a condition known as a spatial equilibrium. Under these conditions, the number of interactions per unit time is given by $I\sim {N}^{\beta }$, where $N$ is the number of interacting individuals and $\beta =7/6$ (Bettencourt, 2013, 2014; Lobo, et al., 2020; Ortman & Lobo, 2020; Ortman, et al., 2015). Consequently, interactions increase faster than population and exhibit increasing returns to population scale. If one then considers that there is an average outcome of an interaction, across all types that occur during a production process, then the total agricultural production of a group $Y$ should increase according to $Y={Y}_{0}{N}^{\beta }$, where ${Y}_{0}$ is the baseline productivity of a farmer given the available technology and $\beta \cong 7/6$. This relationship between population and output has been observed archaeologically for civic architecture construction rates in Mesoamerica and South America (Ortman, et al., 2020). Here, we examine whether this relationship also applies to communal wet paddy rice production at Angkor.

The value of the scaling coefficient $\beta$, which determines the effect on the output of population size, can be estimated through ordinary least-squares regression of log-transformed estimates of population and productivity for a group of cases. This is feasible because $Y={Y}_{0}{N}^{\beta }$ and $\mathrm{log}Y=\mathrm{log}{Y}_{0}+\beta *\mathrm{log}N$ are equivalent expressions, and the latter is a simple linear function.

### Estimating Net Proceeds from Agriculture

One would expect much of the agricultural output of a temple community to have been used to provide subsistence for the people of that community. Inscriptions indicate that a portion of any remaining surplus would also have been transferred to state control in the form of taxation or payment in kind (Lustig, 2009). After that, much of the remaining surplus would have been devoted to construction and maintenance of the community temple and the activities that took place there. We thus consider the total volume of the temple platform(s) in a community as a proxy for the average net proceeds from agriculture in that community, after taking subsistence and transfers into account. These net proceeds were likely only a small percentage of total temple production, and we test this hypothesis as a cross-check on our output measure and as a means of gauging the overall economic fortunes of temple communities. We use the relationship between agricultural outputs and associated temple platform volumes to estimate the average profitability of agriculture for temple communities, with the slope of the best-fit line relating these two log-transformed measures reflects the average percent profit (see “Results,” below).

### Identifying Spatial Patterns in Land Use

Given the extensive area of Angkor and the fact that food surpluses ultimately had to reach the non-food-producing population in the urban core, transport costs were a potentially important factor in deciding where and how intensely to engage in agricultural activities within the settlement area. This specific decision can be further conceived as part of a higher-level decision regarding the type of use to which a given plot of land was put (agriculture, housing, a temple, transport or hydraulic infrastructure, etc*.*). In urban economics, the primary model of land use within cities is the Alonso-Muth-Mills (AMM) model (Alonso, 1964; Brueckner, 1987; O’Sullivan, 2011:125–200), itself an elaboration of Von Thunen’s analysis of agricultural production surrounding a central market (von Thünen, 1966). This model hypothesizes that the increase in transport costs with distance from a central market is counterbalanced by a decrease in land values (land rents in the language of economics).

As with SST, the AMM model builds from the assumption of a balancing of spatial costs and benefits, thus implying that increasing desirability of a location is counteracted by increasing cost (Alonso, 1964; Brueckner, 1987). Under these conditions, moving a residence toward or away from a central work area will change the commuting cost by the change in distance $\Delta x$ times the transport costs per unit distance $\varepsilon$. This change will be balanced by a corresponding change in the value of housing per unit area $\Delta p$ times the area of the house $a$ (O’Sullivan, 2011:141). This can be expressed as:

1 $$\Delta p*a=-\Delta x*\varepsilon .$$

We adapt this relationship to the agricultural landscape of Angkor by viewing $p$ as the value of agricultural land, which we estimate as to its productivity per hectare, and $a$ as the area cultivated by an individual farmer.

Notice that the left side of Eq. (1) can also be expressed as $\Delta a*p$, leading to a system of equations:

2 $$\frac{\Delta p}{\Delta x}=-\frac{\varepsilon }{a},\mathrm{ and }\frac{\Delta a}{\Delta x}=-\frac{\varepsilon }{p},$$

where the left sides of both equations represent the slope of the relationship between land productivity and distance, and the relationship between area per farmer and distance, respectively. Since transport costs $\varepsilon$ are constant, one can solve 2 for $\varepsilon$ and equate them:

3 $$-a\left(\Delta p/\Delta x\right)=-p\left(\Delta a/\Delta x\right),$$

leading to:

4 $$a=\left[\frac{\Delta a/\Delta x}{\Delta p/\Delta x}\right]*p,$$

where the term in brackets is itself the slope of the relationship between area per farmer $a$ and productivity per hectare $p$. Thus,

5 $$\left(\Delta a/\Delta p\right)=\left[\frac{\left(\Delta a/\Delta x\right)}{\left(\Delta p/\Delta x\right)}\right].$$

Equation (5) can be empirically examined using a regression framework. We use the data for temple communities during Period 6, estimating $a$ as temple territory / temple population, $p$ as temple productivity / temple territory, and $x$ as the straight-line distance of that temple from the Bayon temple in the center of Angkor Thom.

## Results

### Relationships Between Community Size and Agricultural Outputs

As discussed above, we used measurements, chronological assessments, locations, and orientations of archaeological features to estimate water management infrastructure, associated lands, total agricultural output, and net proceeds for temple communities in the AMA. Table 4 summarizes the resulting dataset for temple communities established during each period and for all temple communities for which we have complete data that were occupied during each period.

**Table.4:** Summary of the temple community dataset from the AMA. Data takes purchasing into account and only includes temples with complete data (a predicted date from Klassen et al. (2018) and associated occupation mounds). For estimates of the total population of Angkor, see Klassen et al. (2021)

Period	Begin date	End date	Duration	Temple community population	New communities	Mean community population	Largest community population	Associated farming area (km^2^)	New communities/year	Farming area/capita (ha)	Dated temples
A. Data for temples dated to each period											
1	770	906	136	24,894	93	366	3973	1327	0.68	5.33	156
2	907	961	54	33,310	108	444	4799	742	2.00	2.23	164
3	962	1001	39	95,036	212	534	9851	711	5.44	0.75	282
4	1002	1113	111	74,462	173	528	10,781	310	1.56	0.42	216
5	1114	1180	66	31,351	77	514	4036	208	1.17	0.66	90
6	1181	1308	127	4427	7	632	3550	12	0.06	0.27	13

Period	Begin date	End date	Duration	Temple community population	Inhabited communities	Mean community population	Largest community population	Associated farming area (km^2^)	Temple community population growth	Farming area growth (ha/yr)	Area of the urban core (km^2^)
B. Cumulative data for all inhabited temples, assuming continuous occupation											
1	770	906	136	24,894	93	366	3973	1327			39.47
2	907	961	54	55,062	192	402	4799	1321	.0147	− .00009	48.15
3	962	1001	39	136,912	380	455	9851	1279	.0234	− .00084	67.89
4	1002	1113	111	184,275	511	456	10,781	1244	.0027	− .00025	76.91
5	1114	1180	66	209,357	568	466	10,781	1323	.0019	.00093	75.06
6	1181	1308	127	213,521	571	471	10,781	1323	.0002	.00000	79.98

The data summarized in Table 4 shows that the rate of construction of new temples was very fast during the tenth century but slowed to a fraction of a percentage point after that. The table also suggests the average population size of a temple group was in the low hundreds and that this average did not change appreciably over time. Finally, the table suggests the total population of farmers represented by our sample increased from a minimum of around 17,000 in the early tenth century to a minimum of over 200,000 by the early thirteenth century. Note, however, that this sample only includes temples where we have complete data. Klassen et al. (2021) provide a comprehensive accounting of the population of greater Angkor, which suggests the total population of the AMA was at least 500,000 by the thirteenth century.

**Fig. 5:**
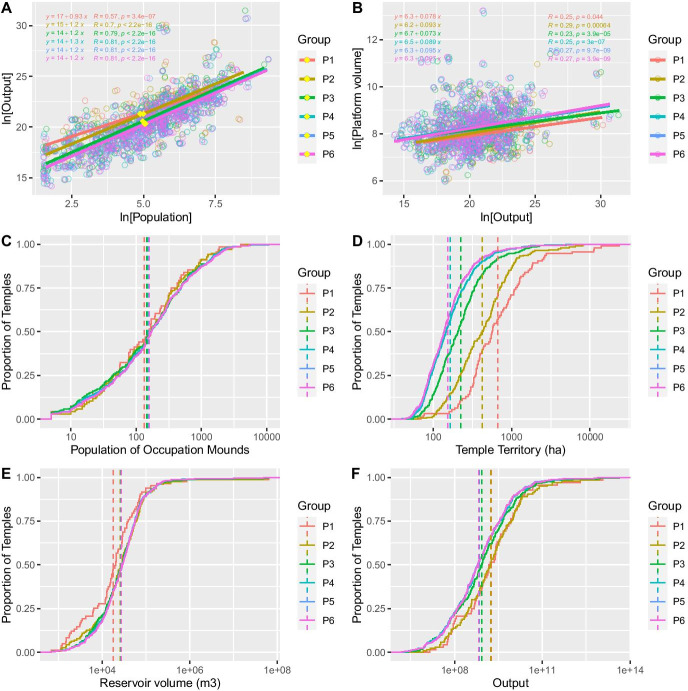
Analysis results by for temple communities inhabited during each chronological period (group), assuming cumulative occupation; **A** temple population vs. agricultural output, yellow diamonds mark the centers of the data for each period; **B** agricultural output vs. temple platform volume, the slope of the fit line reflects net surplus; **C**–**F** cumulative distributions with mean values as dashed lines for: **C** temple populations, note the slight increase in the mean; **D** associated land, note the substantial contraction; **E** irrigation storage, note the increasing trend; and **F** agricultural output, note that declines are modest relative to the reduction in land

Figure 5A uses scaling analysis to estimate the intercept ${Y}_{0}$ and slope $\beta$ of the relationship between population and our output measure for all temple communities occupied during each period. Statistical results for these same data, pooled across periods, are presented in Table 5. In this analysis, labor input is the sum of the residential populations of occupational mounds associated with each temple. The agricultural output measure is the product of the labor, land, and irrigation water associated with that temple, as discussed above. Figure 5A shows that total community outputs increased faster than the population during all but the initial period. This is indicated by a slope greater than one, implying that production per farmer increased with community size on average, with a value reasonably close to that predicted by SST ($\beta =7/6$).

**Table.5:** Analysis results based on characteristics of temple communities occupied during Period 6. All values were log-transformed prior to analysis

Independent	Dependent	Intercept (*SE*)	Slope (*SE*)	*R*^2^	*F*-ratio	*P*-value
Community population	Agricultural output	14.311 (.121)	1.227 (.023)	.611	*F* = 2846 (*DF* = 1,1810)	< 2.2e-16
Agricultural output	Temple platform volume (m^3^)	6.487 (.160)	.085 (.008)	.062	*F* = 120 (*DF* = 1,1810)	< 2.2e-16
Distance*	Output/ha	17.643 (.554)	− .846 (.202)	.037	17.54 (*DF* = 451)	3.37e-05
Distance*	Reservoir volume (m^3^)/ha	6.794 (.306)	− .409 (.104)	.051	27.2 (*DF* = 1,505)	2.67e-07
Distance*	Farming area/person (ha)	− 1.502 (.409)	.521 (.149)	.026	12.22 (*DF* = 1,451)	.00052
Output/ha	Area/person (ha)	8.537 (.346)	− .562 (.022)	.586	637.3 (*DF* = 1,451)	< 2.2e-16
Distance*	Temple platform volume (m^3^)/person	2.754 (.404)	.162 (.147)	.003	1.212 (*DF* = 1,451)	.276

^*^Calculated from the center of Bayon temple in Angkor Thom

**Fig. 6:**
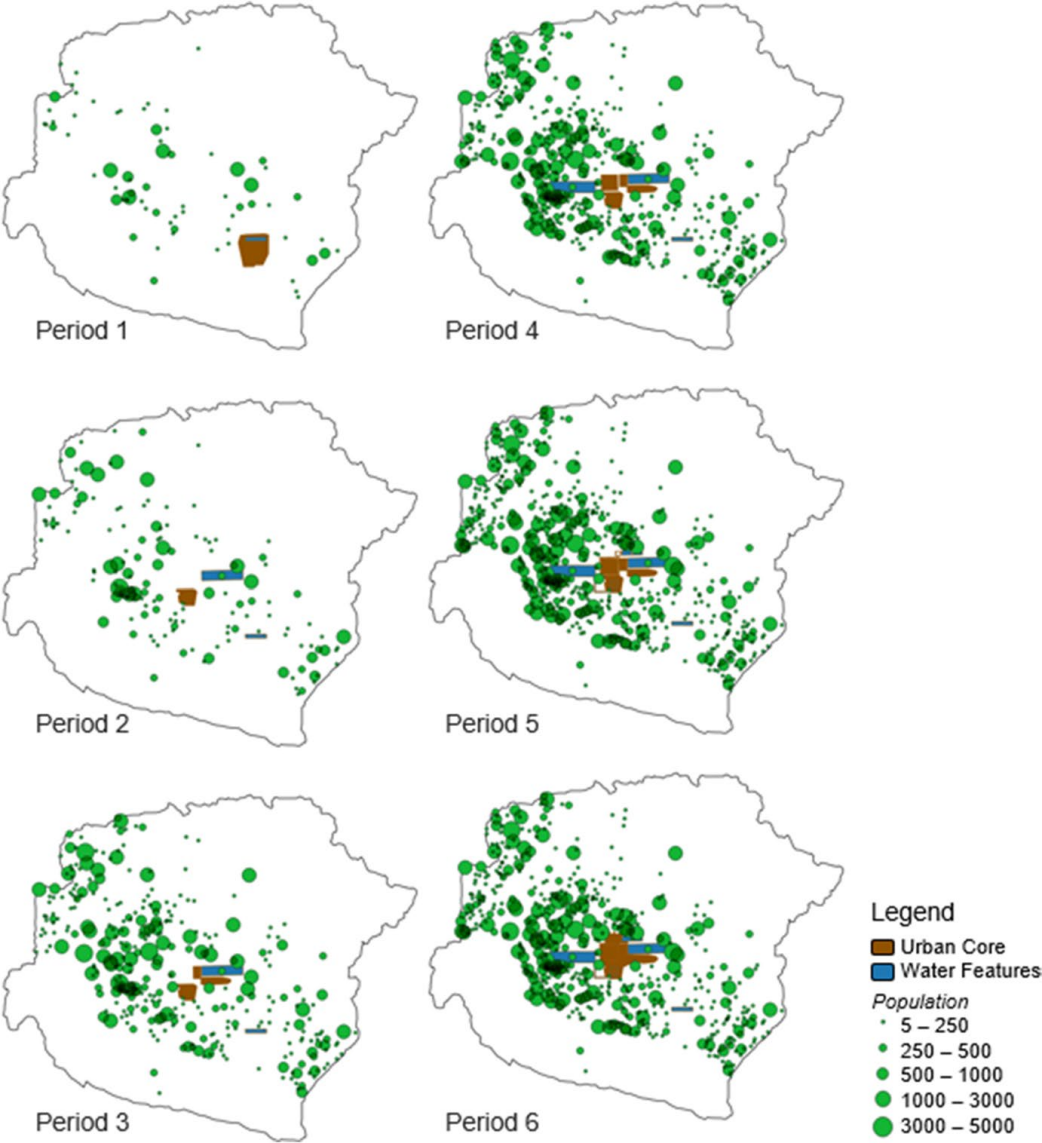
Distribution of temple communities across Angkor by period. Note that the overall extent of temples increased more slowly than the number of temples, such that the primary change through time was increased density of settlement. Despite increasing regional density, temple community yields appear to have sustained increasing returns to labor

Figure 5C–F presents cumulative distributions and group means for production factors through time to characterize this result better (also see Table 5). These charts illustrate that temple territories decreased substantially over time, but temple populations and reservoir storage increased slightly. Figure 6 displays the distribution of temples and their associated populations during each period.

The increases in labor and water inputs (Fig. 5C–F) appear to have counterbalanced decreasing farm size such that temple outputs declined only modestly. Further, the time series mapping (Fig. 6) illustrates the increased packing of temples and population across the Angkor landscape over time. Interestingly, we do not see an expansion of the urban space but rather an infilling over time with occupation in the areas of the AMA closest to the civic-ceremonial centers becoming increasingly dense over time. These results show that agricultural production at Angkor likely exhibited increasing returns to labor at the level of the temple community, at levels that are characteristic of other non-agricultural forms of production in cities.

The scaling relationship parameters (capturing the effects of population size) in Table 5 can illustrate the effect of social organization on agricultural productivity at Angkor. The value ${e}^{14.31}=1.64\times {10}^{6}$ represents the prefactor of the scaling relationship and is an index of the amount produced by a single farmer working alone. The total production of the average-sized temple community, in contrast, is the value of this intercept times the mean community population raised to 1.23 power. So, for a community of 500 persons, the estimated total output is $3.42\times {10}^{9}$, or $6.85\times {10}^{6}$ per person. As a result, per capita, labor productivity in a temple community of 500 persons was $6.85/1.64=4.18$ times that of an individual farmer working alone. This increase in productivity would have been more than adequate to feed a growing population of non-food producers.

Figure 5B also uses regression to estimate the relationship between agricultural output and net proceeds, as proxied by temple volume. Table 5 presents results for these same data pooled across periods. These results suggest that the net proceeds from agriculture varied between about 7 and 10%, with a long-term average of about 8%. This finding suggests that, in addition to benefitting from the state’s services (public events and performances, ritual leadership, protection in warfare, etc*.*), temple communities at Angkor profited directly from communal agricultural production at levels that compare favorably to firms in contemporary economies.

### Spatial Patterns in Land Use

**Fig. 7:**
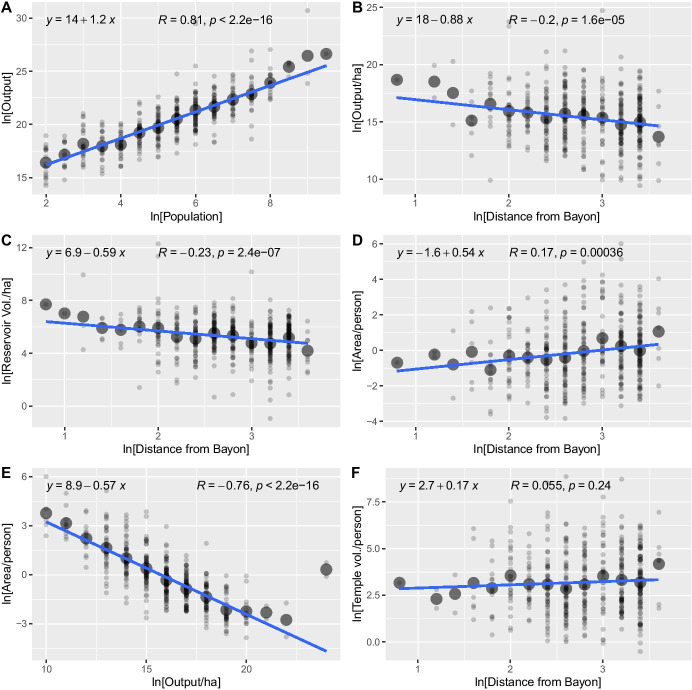
Analysis of urban land use for temple communities during Period 6, based on distance (in km) to the urban core: **A** temple output vs. population; **B** temple output vs. distance; **C** temple reservoir volume (m^3^)/ha vs. distance; **D** land area per person (ha) vs. distance; **E** land area per person vs. output; **F** temple surplus (platform volume, in m^3^) vs. distance. The data are binned to show the relationships more clearly. **A** is binned in .5 unit increments, **B**–**D** and **F** are binned in .2 unit increments, and **E** is binned in 1 unit increments. The larger circles are the mean value of each bin. The AMM model predicts the slope of *F* = 0 and that slope of *E* = (slope of *D* / slope of *B*)

An assessment of the AMM model using data for agricultural temple communities at Angkor is presented in Fig. 7 and Table 5. The regressions in Fig. 7 utilize binned data, which removes some of the noise and illustrates the relationships more clearly, and the regressions in Table 5 use the original, un-binned data. The two sets of results are nearly identical. Figure 7A shows the relationship between output and population for all temple communities inhabited during Period 6, regardless of location. Figure 7B–D shows the effects of distance for output per hectare, reservoir storage per hectare, and land area per person, across temple communities.

Declining productivity with distance appears to relate partly to reduced access to state irrigation infrastructure, with an implied corresponding increase in precipitation-based rice farming. In contrast, increasing area per person with distance likely reflects decreasing land values due to transport costs. Equation (5), above, states that $\left(\Delta a/\Delta p\right)=\left[\left(\Delta a/\Delta x\right)/\left(\Delta p/\Delta x\right)\right]$ where each of the derivatives is the slope of the best-fit line describing the relationship between each pair of variables. Estimates and confidence intervals for these slopes are included in Table 5. These allow us to estimate:

$$\left[\frac{\Delta a/\Delta x}{\Delta p/\Delta x}\right]=\left[\frac{0.521\pm .149}{-0.846\pm .202}\right]=-0.616\pm .351.$$

Due to additive uncertainty, the 95% confidence interval for this ratio is quite broad. Nevertheless, the point estimate is quite close to the estimate for $\left(\Delta a/\Delta p\right)=-.562\pm .022$, illustrated in Fig. 6E, it is impossible to reject the null hypothesis that the two estimates derive from a single population parameter (Student’s *t* = 0.153, *DF* = 900, *P* (1-tailed) = 0.439). Figure 7F and Table 5 also show that temple surplus per capita is independent of distance (the regression is not significant), consistent with the existence of a spatial equilibrium or a balance of costs and benefits at each location in space.

Across these results, distance only accounts for a small percentage of the total variance in the dependent variable. This is likely due to the combined effects of measurement error, time-averaging, the fact that movement was not focused on a central point, and various local conditions affecting land use. Nevertheless, the regressions are significant and show that mean temple productivity and irrigation inputs decrease with distance from the central core as area per farmer increases.

In sum, these results provide multiple lines of evidence supporting the inference that spatial properties of temple communities at Angkor reflect a spatial equilibrium of costs and benefits, both at the level of the agricultural community within which production occurred and at the level of the metropolitan area over which people and food moved. The former accords with SST models of agglomeration effects, and the latter follows the AMM model of urban land use. In addition, the fact that our estimates of land, labor, irrigation inputs, and net proceeds lead to internally consistent results with both sets of models reinforces our methods for translating the archaeological data into proxy measures for these quantities and the overall validity of these proxy measures.

## Conclusion

The results presented in this study suggest that several aggregate properties of the built environment of Angkor are emergent properties of a spatial equilibrium at multiple scales. First, there were increasing returns to scale in the outputs of agricultural temple communities rooted in the spatial concentration of farmers and their interactions that occur when they arrange themselves to balance costs and benefits. This means that larger communities produced more food per farmer, and these surpluses would have been available to feed non-food producers in the urban core. This result contrasts with much previous literature on agricultural intensification, which has presumed that increasing population necessarily leads to decreasing returns. Instead, our results indicate that by increasing the scale of organization of production, an agricultural system can increase output per farmer as regional population density increases. Such increasing returns to scale have been noted for non-agricultural forms of production in both contemporary and ancient systems (Bettencourt, et al., 2007; Glaeser & Gottlieb, 2009; Lobo, et al., 2020). These results also fit within a growing body of literature arguing that intensification should be considered in the context of both spatial and social units (Morrison, 2007) as well as the management of these systems at a supra-family organizational level (Lansing et al. 2017) or as driven by the demands of elites (Morrison, 2006). Although the analytical and modeling framework of increasing returns to labor is often associated with the study of modern and market economies, there is nothing in the conceptual underpinning of increasing returns that restricts its occurrence to market economies.

Second, we have identified spatial patterns in the properties of temple communities that are consistent with the existence of a spatial equilibrium between land values and transport costs at the scale of the metropolitan area. Previous studies of agricultural intensification have focused on production itself, but in urban systems, agricultural outputs also need to travel from production to consumption locations, and there are costs associated with this movement. As a result, agricultural production patterns are not merely based on the availability of land and infrastructure but also the costs of transporting outputs to consumers. For example, Temin (2012) has argued for the existence of a regional wheat market in the Roman Empire based on patterns in the price of wheat with distance from Rome, and Stoner (2017) has also noted the effects of distance for the distribution of field areas surrounding Prehispanic Mesoamerican centers on the Gulf Coast of Mexico. Our results add to this literature, emphasizing the fundamental importance of physical distance, specifically the incurred costs (effort, time) in structuring land-use decisions, including agricultural production.

In addition to offering a new perspective on agricultural intensification, these results contribute to the evolving debate about whether Angkor’s massive hydraulic infrastructure was used to enable multi-cropping. Our results are entirely consistent with the interpretation that water was stored and made available for irrigation, and we find no compelling arguments against this longstanding hypothesis (Evans, 2007). Notwithstanding the absence, at this moment, of conclusive evidence that the barays were used for irrigating wide areas, it is nonetheless clear that the medieval Khmer invested significant energy in creating a vast water storage and distribution system across the Angkor plain and that this water was used for a wide array of “symbolic” and “functional” purposes (Pottier, 2000).

Given our observation of increasing returns, and the scale of the state hydraulic infrastructure, it may be surprising that temple community populations were generally small, with a mean of about 500, and very few that were larger than a few thousand (Fig. 5C). The fact that production groups were modest in size and increased only slightly over time suggests that there were factors other than the supply of stored water that limited the scale of the organization of agricultural labor. It may be that pedestrian movement costs are responsible. Very few temple territories have a transverse dimension larger than 4 km (reflected in the area estimates in Fig. 5D), even though travel costs play no role in their definition. This distance can be comfortably traversed in about 30 min of walking and represents a cross-cultural regularity in commute times to fields and workplaces (Chisholm, 1979; Marchetti, 1994). The hinterlands of agricultural villages in many parts of the world have similar dimensions and populations, but their agricultural production systems vary substantially. Angkor appears to represent a system that made it feasible for village-sized agricultural groups operating within territories of limited size to be highly productive and to maintain increasing returns to scale even in the face of increasing regional population density.

One of the most obvious and robust empirical regularities of modern cities is the significant spatial variation in the intensity of urban land use. Given this, it is notable that, despite relatively low density overall, Angkor exhibits internal spatial variation in land use similar to that seen in modern cities (Fletcher, 2009). The prevalent explanatory framework for such patterns in urban economics—the Alonso-Mulls-Muth model—posits that commuting cost differences within an urban area must be balanced by differences in the price of living space (Brueckner, 1987). Desirable proximity to the urban core entails greater demand on land, making it more expensive. In the contemporary setting, land is an input into the production of housing, infrastructure, or commercial establishments. In the case of Angkor, inhabitants chose between using land as an input into agriculture and some other use, including urban residence, civic-ceremonial activity, transport, and hydraulic infrastructure. We find multiple lines of evidence that transport costs were balanced against the potential value of agricultural land. This spatial pattern exemplifies one of the central theoretical constructs of economic geography and urban economics, spatial equilibrium, and is simply explained by noting that if something is particularly good in one specific location, then we should expect to find something negative in the same place which partially or completely offsets the locational advantage (Glaeser, 2008). Thus, the internal structure of Angkor, a prototypical example of low-density agrarian urbanism, seems to have nevertheless resulted from underlying mechanisms that are common to contemporary cities. Further, the combination of the spatial patterning of the urban landscape and the demonstrated increasing returns to labor indicate that agricultural production was incorporated in the urban economy.

An important question that we are not able to answer using current evidence is the extent to which the agricultural economy of Angkor involved exploitation vs. shared prosperity. Agricultural intensification could have involved the coercive subjugation of farmers, as scholars from Wittfogel (1957) to Scott (2017) have argued. Still, it could also have emerged through more collective institutions involving a communal organization of agricultural production, extensive investment in *landesque capital* (rice paddies and reservoir embankments), management of hydraulic infrastructure, and a division of labor in production (Blaikie & Brookfield, 1987).

Although our findings suggest that temple communities generated increasing returns, this analysis does not allow us to determine the distribution of these returns among individual farmers or the extent to which it benefited them directly. It is quite possible that a small local elite siphoned off all surplus, after providing subsistence wages to tenant laborers and transferring the appropriate share to the state, such that the average farmer benefitted only indirectly from being part of the Angkor state, with static or even decreasing material living standards. On the other hand, it is also possible that temple communities were organized more communally, in which case the temple would have been a symbol of shared surplus directed to shared benefits for the entire community. Future research could focus on obtaining the necessary data for distinguishing between these two hypotheses. The development of Angkor may have led to the immiseration of farmers, but our analyses suggest that, if this was the case, it was not due to decreasing per capita outputs, as often occurs with intensification in smallholder systems. Regardless, the important conclusion from this work is that agricultural production’s social and spatial organization are important and that even with static technology and work hours, per capita productivity can increase through the scale of social organization of production, which appears to have played an important role in the food system of Angkor. Based on these results, we suggest increased attention to these factors in studies of the agricultural economies of other early urban systems.

## Supplementary Information

Below is the link to the electronic supplementary material.

Supplementary file1 (DOCX 97 KB)

## Data Availability

The temple data used in this analysis available at https://core.tdar.org/project/392021/social-reactors-project-datasets. The digital terrain model of the Greater Angkor Metropolitan Area is courtesy of the Khmer Archaeology LiDAR Consortium.
