# Supplementary material for: Provisioning an Early City: Spatial Equilibrium in the Agricultural Economy at Angkor, Cambodia
Source: J Archaeol Method Theory. 2021 Sep 23;29(3):763–94. doi: 10.1007/s10816-021-09535-5 (PMC9402775; doi:10.1007/s10816-021-09535-5)
Supplement: Supplementary file 1 — Supplementary file1 (DOCX 97 KB) [file 10816_2021_9535_MOESM1_ESM.docx]

**Increasing returns to agricultural intensification at Angkor, Cambodia**

Sarah Klassen^1*^, Scott G. Ortman^2^, José Lobo^3^, and Damian Evans^4^

**Author Information**

^1^Department of Anthropology, University of British Columbia and Archaeological Sciences, Leiden University

^2^Department of Anthropology and Institute of Behavioral Science, University of Colorado Boulder; Santa Fe Institute

^3^School of Sustainability, Arizona State University

^4^École française d'Extrême-Orient, 22 Avenue du Président Wilson, 75116 Paris, France

Contributions

SK, SO, and JL contributed to the planning of this research; SK, SO, and JL drafted the manuscript; SK, SO, JL and DE edited the manuscript, SK, SO, and JL contributed to the analysis; SK and SO compiled the data; SK contributed data; SK and SO designed the figures. All authors discussed the results and commented on the manuscript.

Corresponding author

Sarah Klassen (https://orcid.org/0000-0001-5110-713X)

**Supplementary Information**

***Ethnographic evidence for increasing returns from irrigated rice production:*** Figure S1 presents ethnographic data collected from *communidades* in the Indian state of Goa, ca. 1965 (1). The population measure is the number of tenants who worked the rice paddies in a community, the area measure is the hectares of rice paddy in production in that community, and the output measure is the khandi (1 khandi = 266 pounds) of rice produced by that group per year. The two data series show that communidades with more tenants produced greater output per person, even as they cultivated fewer hectares per person. These data provide more direct documentary evidence that community-scale organization in agricultural production can generate increasing returns to labor and are generally consistent with the archaeological results from Angkor discussed in the main text.

Figure S1: Relationships between farming labor, land, and outputs in *communidade* rice paddies in Goa, India, ca. 1965. Data are from Axelrod and Fuerch 2006: Table 2.


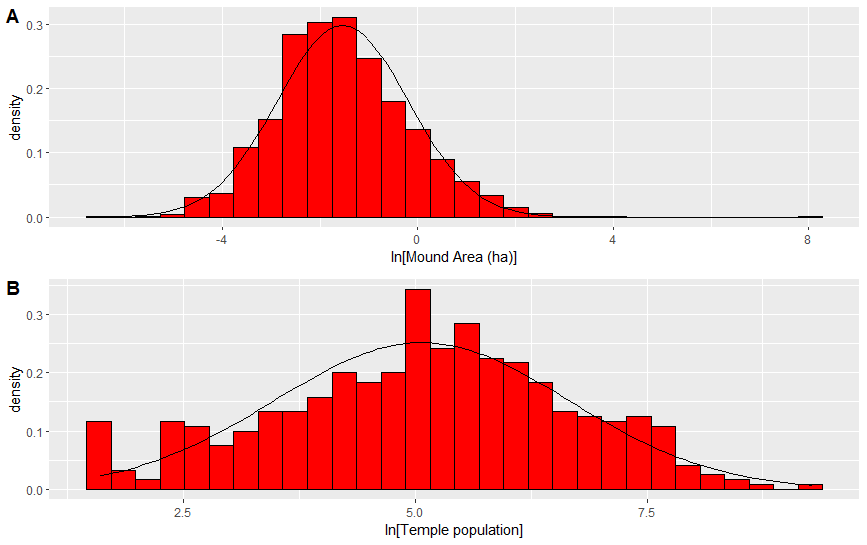


Figure S2: Distribution of occupation mound areas and resulting temple community populations across the agricultural hinterland of Angkor: A, mound areas; B, population estimates during Period 6, based on the amorphous settlement model, and after taking purchasing into account. Note that both distributions exhibit reasonable fits to log-normal distributions. The lower tail of mound areas is somewhat compressed, most likely due to biased destruction of smaller mounds; and jaggedness in the lower tail of temple community populations is due to the use of a minimal residential population for very small mounds. Based on this analysis, the mean population of temple communities is 471, and the largest temple community population is 10,780.


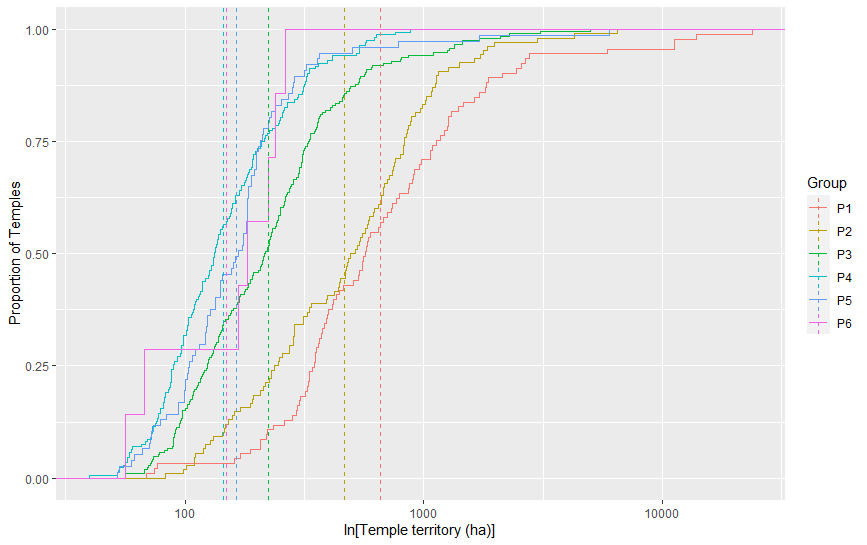


**Figure S3. Cumulative distributions of new temple territories, by dedication period. Dashed lines represent the mean temple territory for each period.**


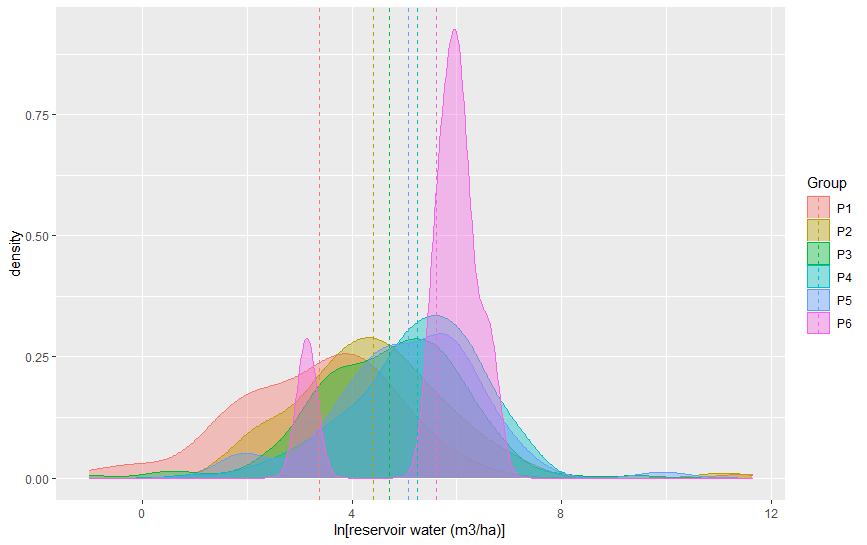


**Figure S4. Density plots for reservoir water (m^3^ per hectare of land), by construction period of the associated temple, assuming reservoirs were built at the same time as agricultural temples. Dashed lines represent group means.**


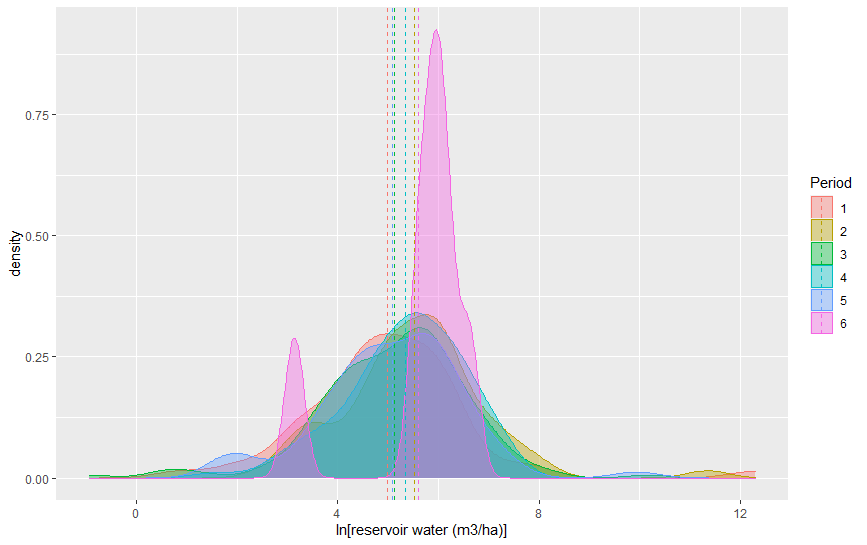


**Figure S5. Density plots for reservoir water (m^3^ per hectare of land), by construction period of the associated temple, assuming preserved reservoirs reflect conditions on the landscape during the final period. Dashed lines represent group means.**


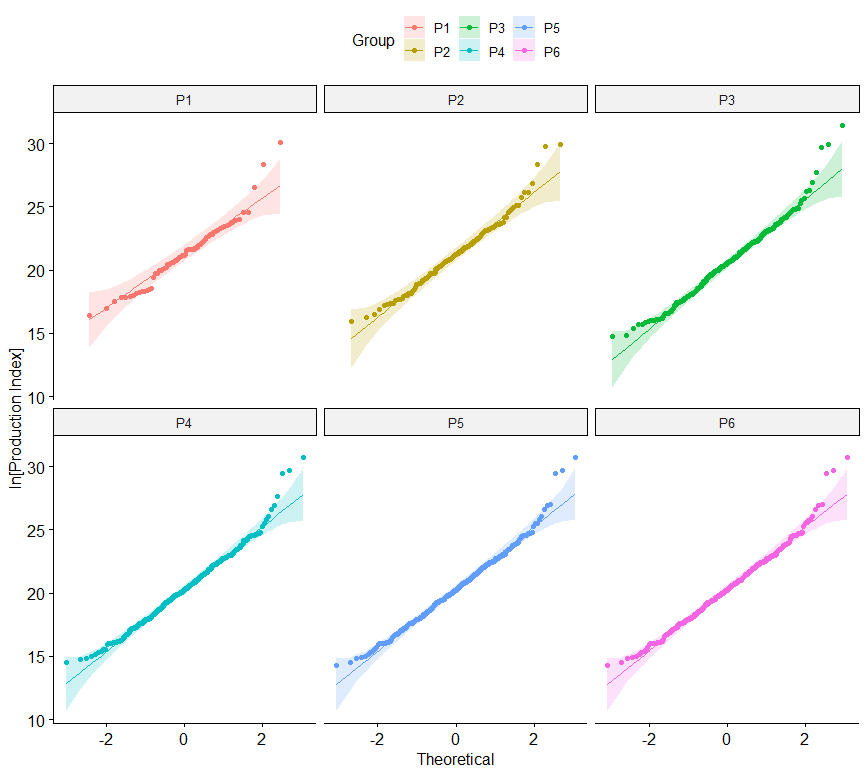


Fig. S6. QQ plots of temple community production indices, by period.

Table S1. Depth Regressions for features in the Angkor Metropolitan Area.

|  | Total | R^2^ | Regression equation | Total features within the Greater Angkor region |
| --- | --- | --- | --- | --- |
| Reservoir | 6161 | 0.027 | y = -0.0873 + 0.263*ln(*x*) | 9,876 |
| Reservoir embankment | 509 | 0.325 | y = 0.377 + 0.199(*x*) | 2,817 |
| Moats | 224 | 0.3459 | y = -2.43 + 0.578*ln(*x*) | 922 |
| Temples | 150 | 0.043 | y = 1.43 + 0.0000685(*x*) | 1009* |

*Of these, 921 temples are in the Greater Angkor region and have predicted dates.

Table S2: Features in the civic-ceremonial zone and the Angkor Metropolitan Area.

|  | AMA | Civic-ceremonial zone |
| --- | --- | --- |
| Reservoirs  *Total Count*  *Total Volume* | 4,625  16.16 km^3^ | 5,253  0.28 km^3^ |
| Moats  *Total Count*  *Total Volume* | 789  0.02 km^3^ | 133  0.004 km^3^ |
| Temples  *Total Count*  *Total Area* | 749  2.09 km^2^ | 262  0.89 km^2^ |

**Table S3: Reservoirs and moats that were grouped with temples in the Angkor Metropolitan Area.**

|  | Temple | No temple |
| --- | --- | --- |
| Reservoirs  *Total Count*  *Total Volume / Area* | 2163  16.0 km^2^ | 2462  0.16  km^2^ |
| Moats  *Total Count*  *Total Volume / Area** | 687  0.0055 km^2^ | 102  0.0144km^2^ |

**References**

1. Axelrod P & Fuerch M (2006) Common Ground: Risk, Scarcity, and Shared Resources in Goan Agriculture. *Human Ecology* 34(1):79-98.
